# Mannitol as a crystalloid substitute for colloid oncotic support in the respiratory active working heart–brainstem preparation

**DOI:** 10.1016/j.crphys.2026.100192

**Published:** 2026-10-20

**Authors:** Ahmet Oguzhan Bicakci, Swen Hülsmann

**Affiliations:** Department of Anesthesiology, University Medical Center, Georg-August University, Humboldtallee 23, Göttingen, 37073, Germany

**Keywords:** Working heart–brainstem preparation, Mannitol, Ficoll, Perfusate, Edema, Phrenic nerve, Respiratory rhythm, preBötzinger complex

## Abstract

The working heart–brainstem preparation (WHBP) is an arterially perfused in situ rodent model that requires an osmotically or oncotically active perfusate additive to limit edema and maintain stable central respiratory motor output. Ficoll-70 at 1.25% (w/v) is widely used for this purpose, but at this concentration it generates only modest colloid osmotic pressure (∼3–4 mmHg) and presents practical limitations, including batch variability, endotoxin risk, and high cost. We asked whether mannitol at 0.85% (w/v), a small-molecule crystalloid osmolyte and hydroxyl-radical scavenger with an established clinical safety profile, can substitute for Ficoll-70 while preserving WHBP viability.

Adult mouse WHBPs (n = 7 Ficoll; n = 9 mannitol) were perfused for 1 h with Krebs-based perfusate containing either 1.25% Ficoll-70 or 0.85% mannitol. Preparations were assessed for phrenic and vagus nerve activity, perfusion pressure, whole-preparation weight gain, brain tissue weight, and single-neuron recording capability. Phrenic discharge frequency increased over time in both groups (Ficoll: 0.15 → 0.49 Hz; mannitol: 0.24 → 0.45 Hz), and amplitude of the integrated phrenic burst did not differ significantly between additives. Perfusion pressure rose modestly over 1 h (Ficoll: 67.7 → 76.7 mmHg; mannitol: 67.2 → 74.1 mmHg), with no significant between-group difference. Whole-preparation weight increased in both conditions, indicating tissue water uptake during perfusion. Brainstem–cerebellum wet weight after 1 h was similar with both perfusates (0.140 ± 0.023 g, Ficoll; 0.124 ± 0.020 g, mannitol; p = 0.18). Juxtacellular recordings from preBötzinger complex neurons confirmed that single-unit activity can be obtained reliably in mannitol-perfused preparations for more than 2 h. Moreover, post-inspiratory activity of the vagus nerve is preserved in mannitol-perfused preparations.

Together, these data indicate that 0.85% mannitol maintains WHBP cardiorespiratory function and tissue water balance to a degree comparable with 1.25% Ficoll-70, while providing a lower-cost, endotoxin-free alternative. The use of mannitol as a crystalloid perfusate additive may therefore facilitate wider and more reproducible application of the WHBP and related arterially perfused in situ preparations in integrative physiology and pharmacology.

## Highlights

• Mannitol (0.85% w/v) replaces Ficoll-70 (1.25% w/v) without reducing WHBP stability.
• Phrenic burst frequency and amplitude are indistinguishable with mannitol and Ficoll over 1 h.
• Brainstem–cerebellum wet weight at 1 h is unaffected by the osmotic additive, indicating comparable tissue water balance.
• Mannitol offers a low-cost, endotoxin-free, batch-stable alternative to Ficoll-70 for WHBP studies.

## 1 Introduction

Isolated and in situ organ perfusion preparations occupy a unique and increasingly important position in biomedical research. As regulatory pressure to reduce and replace in vivo animal experimentation intensifies under frameworks such as EU Directive 2010/63/EU and the 3Rs principles, perfused organ and whole-body in situ preparations offer a scientifically rigorous intermediate that can substantially reduce the number of fully conscious animals required for systems-level physiological and pharmacological investigation. Unlike purely in vitro preparations such as brain slices or isolated cell cultures, perfused in situ preparations retain intact neural circuits, sensory receptors, efferent motor nerves, and functional end organs — enabling integrative experiments that are not possible at the cellular level. These preparations, like the working heard brainstem preparation (Paton, 1996a; Paton et al., 2022) are critically dependent on the composition of the artificial perfusate to maintain tissue viability over the course of an experiment.

A central challenge in arterially perfused preparations is the absence of plasma proteins. In the intact organism, plasma proteins, especially albumin, generate a colloid osmotic (oncotic) pressure of approximately 25–28 mmHg that counteracts the hydrostatic filtration pressure at the capillary wall, thereby preventing progressive fluid extravasation into the interstitium (Levick and Michel, 2010). When cell-free crystalloid perfusates are used, this oncotic pressure is absent, and the resulting net outward hydrostatic gradient causes tissue edema within minutes, particularly in highly vascular organs like the brainstem (Paton, 1996a). All arterially perfused in situ preparations must therefore include an osmotically or oncotically active additive to limit this edema and maintain experimental stability. The choice of that additive, a practical decision made by most laboratories without systematic quantitative evaluation, directly determines perfusate cost, reproducibility, and physiological appropriateness.

The working heart–brainstem preparation (WHBP) is one of the most versatile and widely adopted of these in situ preparations (Paton, 1996a; Paton et al., 2022). First described by Paton in 1996, the WHBP consists of a decerebrate rodent perfused retrogradely via the descending aorta with a carbogenated cell-free Krebs solution, simultaneously maintaining an intact brainstem capable of generating a stable eupneic respiratory motor pattern (Paton, 1996a). The preparation is now used in many laboratories worldwide to study respiratory rhythm generation, autonomic cardiovascular control, chemoreception, neuroendocrine regulation, and brainstem–cardiac coupling (Paton et al., 2022). The preparation is also well suited to pharmacological studies, allowing precise control of drug concentration, perfusate composition, temperature, and gas tensions that is not achievable in vivo, a property of direct relevance to neuropharmacology, cardiorespiratory pharmacology, and the testing of autonomically active compounds.

Since its introduction, the WHBP has employed macromolecular colloids as oncotic additives to prevent edema. The original description used industrial-grade dextran; subsequent laboratories have adopted polyvinylpyrrolidone (PVP), sucrose, polyethylene glycol (PEG), and Ficoll-70 (Paton, 1996a; Paton et al., 2022). Of these, Ficoll-70, a high-molecular-weight sucrose-epichlorohydrin copolymer, (MW > 70 kDa), has become the most widely used, because its high reflection coefficient (σ ≈ 1) means it does not readily cross interendothelial clefts and therefore maintains a sustained transcapillary oncotic gradient throughout perfusion. However, Ficoll presents a number of practical and scientific limitations. At the concentrations used in the WHBP (1.25% w/v), Ficoll generates only approximately 3-4 mmHg of oncotic pressure, which is below normal plasma oncotic pressure and substantially less than the oncotic support routinely employed in other isolated perfused organ preparations (Maack, 1980; Schurek and Alt, 1981; Swanson et al., 1981). On the other hand, polysaccharide preparations carry a recognized risk of endotoxin contamination, exhibit significant batch-to-batch variability in molecular weight distribution and oncotic activity, and represent a substantial financial cost (>$30 per litre of perfusate at current catalogue prices) that creates barriers to wider laboratory adoption.

Mannitol, a six-carbon sugar alcohol (molecular weight ≈ 182 Da), is a mechanistically distinct candidate for replacing Ficoll in WHBP perfusate. As a small, membrane-impermeant osmole, mannitol raises perfusate osmolality — at 0.85% (w/v) by approximately 47 mOsm/L — generating an osmotic drive that opposes net transcapillary water flux and attenuates cellular swelling (Kellen and Bassingthwaighte, 2003). In addition, mannitol is a well-characterized scavenger of hydroxyl radicals (Gan et al., 2026; Magovern et al., 1984), the most cytotoxic reactive oxygen species produced during ischemia–reperfusion; this antioxidant property may provide additional protection against oxidative tissue damage during the reperfusion and the reoxygenation phase (95% O_2_; 5%CO_2_) inherent to WHBP establishment. Mannitol is also chemically stable, available at pharmaceutical grade with negligible endotoxin burden, metabolically inert in the tissues retained in the WHBP, and orders of magnitude less expensive than research-grade Ficoll. Consistent with these properties, mannitol is widely used as a hyperosmolar agent in clinical practice to treat intracranial hypertension and cerebral edema (Cook et al., 2020).

Despite the established requirement for an oncotic or osmotic additive in WHBP perfusate, no study has directly compared the functional stability of the preparation, its central respiratory motor output, and tissue water content when Ficoll is replaced by mannitol. The present study therefore tested the hypothesis that 0.85% mannitol, incorporated into the perfusate as a hyperosmolar crystalloid additive, preserves central respiratory pattern generation, perfusion pressure stability, and limits tissue edema to a comparable extent as 1.25% Ficoll in the mouse WHBP.

## 2 Methods

### 2.1 Ethics and animals

Experiments were performed using adult mice (both sexes, 20–41 g). Breeding, housing, and all experimental procedures complied with the European Communities Council Directive 2010/63/EU. The study protocol was approved by the Animal Welfare Commission of the University.

### 2.2 Working heart–brainstem preparation

The working heart–brainstem preparation (WHBP) was established following an established protocol (Paton, 1996b)) and subsequent refinements (Tacke et al., 2023). Briefly, mice were deeply anaesthetized with isoflurane until apnea and absence of nociceptive withdrawal reflex were confirmed. The animal was bisected below the diaphragm for exsanguination, and the forebrain was removed. The descending aorta was prepared and cannulated retrogradely with a tube attached double-lumen catheter and perfused at 20 mL/min using a peristaltic pump (Watson–Marlow). The perfusate was an artificial cerebrospinal fluid (ACSF)-like solution, carbogenated with 95% O_2_/5% CO_2_ and maintained at 32°C (pH 7.4), with the following composition (in mM): 125 NaCl, 24 NaHCO_3_, 2.5 CaCl_2_, 1.25 MgSO_4_, 1.25 KH_2_PO_4_, 5 KCl, and 10 glucose. Perfusion pressure was monitored continuously via the second lumen of the catheter and recorded as aortic perfusion pressure. A total of 16 preparations were included in the analysis: 7 in the Ficoll group and 9 in the mannitol group.

### 2.3 Perfusate additives: Ficoll and mannitol

Preparations were assigned to one of two groups according to the osmotic additive in the perfusate: Ficoll® PM 70 (Sigma–Aldrich, F2878) at 1.25% (w/v) or D-mannitol (Sigma–Aldrich, M9546) at 0.85% (w/v). Ficoll® PM 70 is a high-molecular-weight sucrose-co-epichlorohydrin polymer that behaves as a macromolecular colloid; at 1.25% it generates an oncotic pressure of approximately 3-4 mmHg (van ’t Hoff estimate). D-Mannitol is a small hydrophilic osmole; at 0.85% it contributes ∼47 mOsm/L to perfusate osmolality and is widely used clinically as a hyperosmolar agent. In both groups, the base ACSF composition and perfusion parameters were identical.

### 2.4 Nerve recordings

A thoracic phrenic nerve, and some experiment also the cervical vagus nerve, was dissected and drawn into a custom-made borosilicate glass suction electrode. Phrenic nerve activity (PNA) and was amplified and band-pass filtered (0.25–2 kHz) using a custom-built amplifier (electronic workshop, Department of Physiology), digitized with a PowerLab interface (ADInstruments), and stored on a Windows 10/11 PC for offline analysis.

The phrenic signal digitally filtered (bandstop, 2Hz-500Hz) and integrated in LabChart using the Integral channel calculation (absolute value, time-constant decay, tau = 100-200 ms). The ∫PNA signal was analyzed in LabChart using the Peak Analysis module.

Respiratory frequency (fR; Hz) was calculated as the reciprocal of the mean peak-to-peak interval of the integrated phrenic nerve activity (∫PNA) signal. For the definition of inspiratory duration, burst onset (T_start_) and termination (T_end_) were defined, respectively, as the first and last data points exceeding 20% of the burst height above the automatically determined baseline. Inspiratory duration (T_I_) was defined as T_end,n_ – T_start,n_ and total respiratory-cycle duration (T_TOT_) as T_start,n+1_ - T_start,n._ Inspiratory duty cycle was calculated as T_I_/T_TOT_. The amplitude of the integrated PNA signal (arbitrary units) was normalized to the initial amplitude at the start of perfusion and the perfusion pressure (PP; mmHg) measured at the aortic catheter tip at the beginning of perfusion and after 1 h.

For assessment of single-neuron recording feasibility under mannitol perfusion, juxtacellular recordings were obtained from preBötzinger complex neurons in a subset of mannitol-perfused preparations (n = 4). Glass microelectrodes were positioned using a motorized stereotactic arm and advanced into the preBötzinger complex under visual guidance. Neuronal action potentials were recorded simultaneously with phrenic nerve activity, allowing the respiratory phase relationship of individual preBötzinger complex neurons to be determined.

### 2.5 Statistical analysis

Data are presented as mean ± SD unless otherwise stated. Statistical analyses were performed using GraphPad Prism 9 (GraphPad Software, San Diego, CA, USA). Whole-preparation weight, respiratory frequency (f_R_) and, perfusion pressure (PP) were analyzed using a two-way repeated-measures (RM)-ANOVA, with perfusate additive (Ficoll versus mannitol) as the between-preparation factor and time (before versus after 1 h) as the repeated-measures factor. Šídák's multiple-comparisons test was used for post hoc pairwise comparisons.

For brainstem-cerebellum weight, relative amplitude changes of ∫PNA, inspiratory duration (T_I_) and inspiratory duty cycle between-group differences were compared between groups using Welch's unpaired t-tests. A two-sided p < 0.05 was considered statistically significant.

## 3 Results

### 3.1 Respiratory network activity

We first assessed whether replacement of Ficoll 70 (1.25%) with mannitol (0.85%) altered respiratory network activity in the working heart–brainstem preparation (WHBP).

Respiratory frequency (fR) increased over the first hour of perfusion in both groups, from 0.15 ± 0.10 Hz to 0.49 ± 0.29 Hz in Ficoll-perfused preparations (n = 7) and from 0.24 ± 0.15 Hz to 0.45 ± 0.30 Hz in mannitol-perfused preparations (n = 9; Fig. 1). Two-way repeated-measures ANOVA revealed a significant main effect of time (F_(1,14)_ = 15.91, p = 0.0013), indicating an overall increase in f_R_ as the preparations stabilized. There was neither evidence of a main effect of perfusate additive (F_(1,14)_ = 0.11, p = 0.75) nor an additive × time interaction (F_(1,14)_ = 0.94, p = 0.35). Sidák-adjusted comparisons showed no difference between Ficoll- and mannitol-perfused preparations either at the beginning of perfusion (p = 0.648) or after 1 h (p = 0.937).

**Fig. 1:**
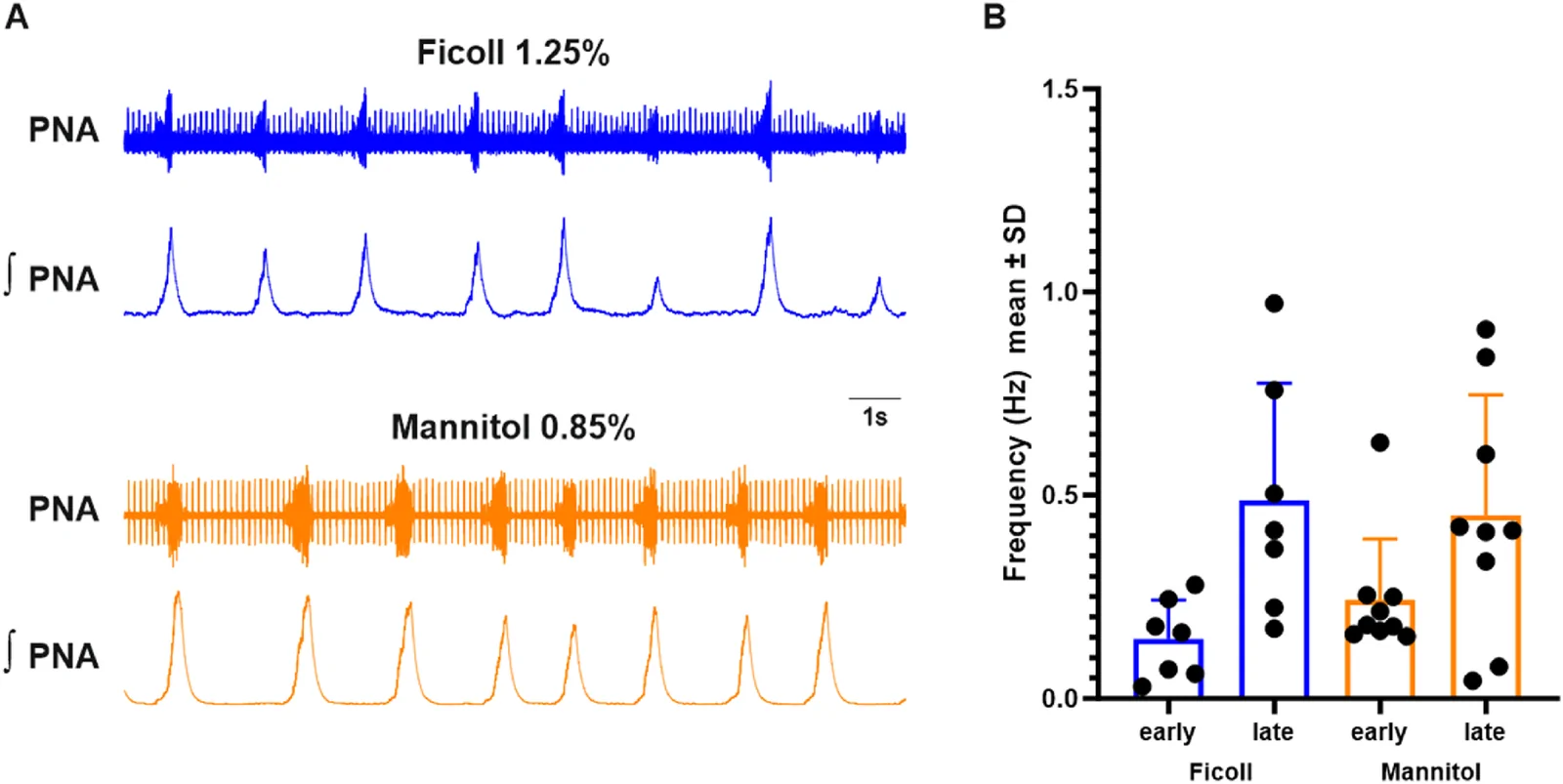
**Comparison of effects of Ficoll and mannitol on the network activity in the working heart brainstem preparation.** A) Representative recordings from the phrenic nerve activity (PNA) with raw and integrated signal in the WHBP after 1 h of perfusion with Ficoll 1.25% and mannitol 0.85%. B) Statistical analysis of the respiratory frequency (Hz) at the beginning and after 1 h of activity, after perfusion with either Ficoll 70 (1.25%) or mannitol (0.85%).

The amplitude of the ∫PNA signal decreased during perfusion in both groups. With Ficoll, ∫PNA amplitude fell to 47.7 ± 27.7% of its initial value (n = 7), whereas with mannitol it remained at 68.3 ± 41.5% of baseline (n = 9). Although the amplitude remained relatively larger in the mannitol group, this difference did not reach statistical significance, indicating that both formulations maintain phrenic discharge amplitude within a comparable range over 1 h (p = 0.2558; Welch's unpaired *t*-test).

Perfusion pressure (PP) at the aortic catheter tip increased over time in both groups. In the Ficoll group, PP rose from 67.73 ± 9.84 mmHg at the start of perfusion to 76.66 ± 18.29 mmHg after 1 h. In the mannitol group, PP increased from 67.21 ± 5.38 mmHg to 74.13 ± 5.75 mmHg. Two-way repeated-measures ANOVA revealed a significant main effect of perfusion time (F_(1,14)_ = 17.14, p = 0.0010). There was neither evidence of a main effect of perfusate additive (F_(1,14)_ = 0.0955, p = 0.7618) nor an additive × time interaction (F_(1,14)_ = 0.2732, p = 0.6094). Sidák-adjusted comparisons showed no difference between Ficoll- and mannitol-perfused preparations either at the beginning of perfusion (p = 0.9939) or after 1 h (p = 0.8681), indicating that vascular resistance increased during the experiment irrespective of the additive used, with no significant difference in PP between Ficoll and mannitol.

### 3.2 Timing of inspiration is not altered in mannitol-perfused preparations

We also analyzed the inspiratory timing after 1h of perfusion. Inspiratory duration (T_I_) did not differ between groups, measuring 1.34 ± 1.09 in Ficoll-perfused preparation (n = 7) and 1.49 ± 0.99 in Mannitol-perfused preparation (n = 9; Welch's unpaired *t*-test, P = 0,78). Similarly, inspiration duty cycle, defined as T_I_/(T_TOT_) was comparable between Ficoll- and mannitol-perfused preparations (0.38 ± 0.19 in Ficoll perfused preparation and 0.40 ± 0.15 in Mannitol perfused preparation; p = 0.84).

### 3.3 Effects of mannitol and Ficoll on WHBP weight

One possible cause of the increase of the resistance is extravasation of water. To test if Ficoll 70 (1.25%) or mannitol (0.85%) are better to prevent extravasation of fluid in the WHBP, we weighted of the preparation just before we started the canulation and after 1h of recording. However, with both substances there was a significant increase of the weight indicating after 1 h perfusion that neither 1.25% Ficoll or 0.85% mannitol are able to prevent extravasation of fluid in the WHBP (Fig. 2). With Ficoll, there was a significant increase of the weight of the preparation from 6.89 ± 1.11 g to 9.41 ± 1.52 g (n = 7). With mannitol the weight of the preparation increased from 7.02 ± 0.65 g to 10.86 ± 1.26 g (n = 9).

**Fig. 2:**
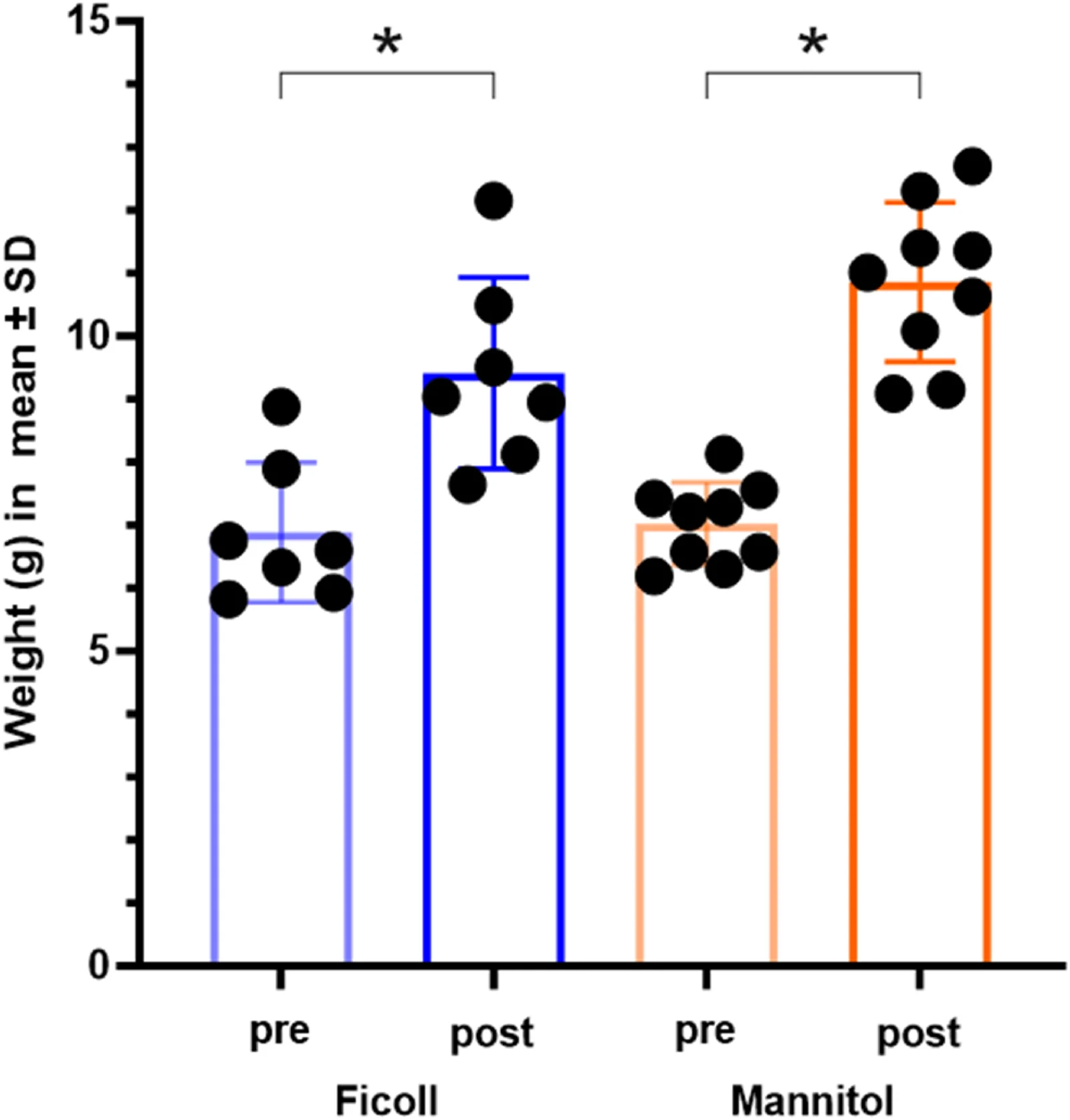
**Comparison of effects of Ficoll and mannitol on the weight of the in the working heart brainstem preparation.** The weight was taken before canulation of the aorta and after 1 h of perfusion. Asterisks represent significant differences (two-way Anova Test).

The RM two-way RM-ANOVA (Ficoll vs. Mannitol × Before vs. After), with perfusate additive (Ficoll versus mannitol) as the between-preparation factor and time (before versus after 1 h) as the within-preparation factor, demonstrated a significant additive × time interaction F_1,14_ = 10.09; p = 0.0067). Thus, the weight gain differed between perfusates. Whole-preparation weights did not differ before perfusion (Sidák-adjusted p = 0.9652); however, after 1 h, Ficoll-perfused preparations weighed less than those perfused with mannitol (estimated marginal mean difference, Ficoll − mannitol: −1.45; 95% CI, −2.82 to −0.07 g; Sidák-adjusted p = 0.0384). Accordingly, weight gain over 1 h was smaller in Ficoll-perfused than in mannitol-perfused preparations.

Whether either additive reduced the absolute extent of edema relative to plain crystalloid perfusate cannot be determined from the present design, as a no-additive control group was not included.

### 3.4 Brain tissue weight

Because the brainstem and cerebellum account for only a small fraction of total preparation mass, we additionally assessed their combined wet weight after 1 h of perfusion. Brainstem-cerebellum tissue wet-weight was 0.124 ± 0.020 g (n = 9) in mannitol-perfused preparations and 0.140 ± 0.023 g (n = 7) in Ficoll-perfused preparations. This difference was not statistically significant (Welch's unpaired *t*-test, p = 0.1903, Fig. 3). Thus, despite lower whole-preparation weight gain with Ficoll, the data provide no evidence of a corresponding difference in swelling-induced brainstem weight gain.

**Fig. 3:**
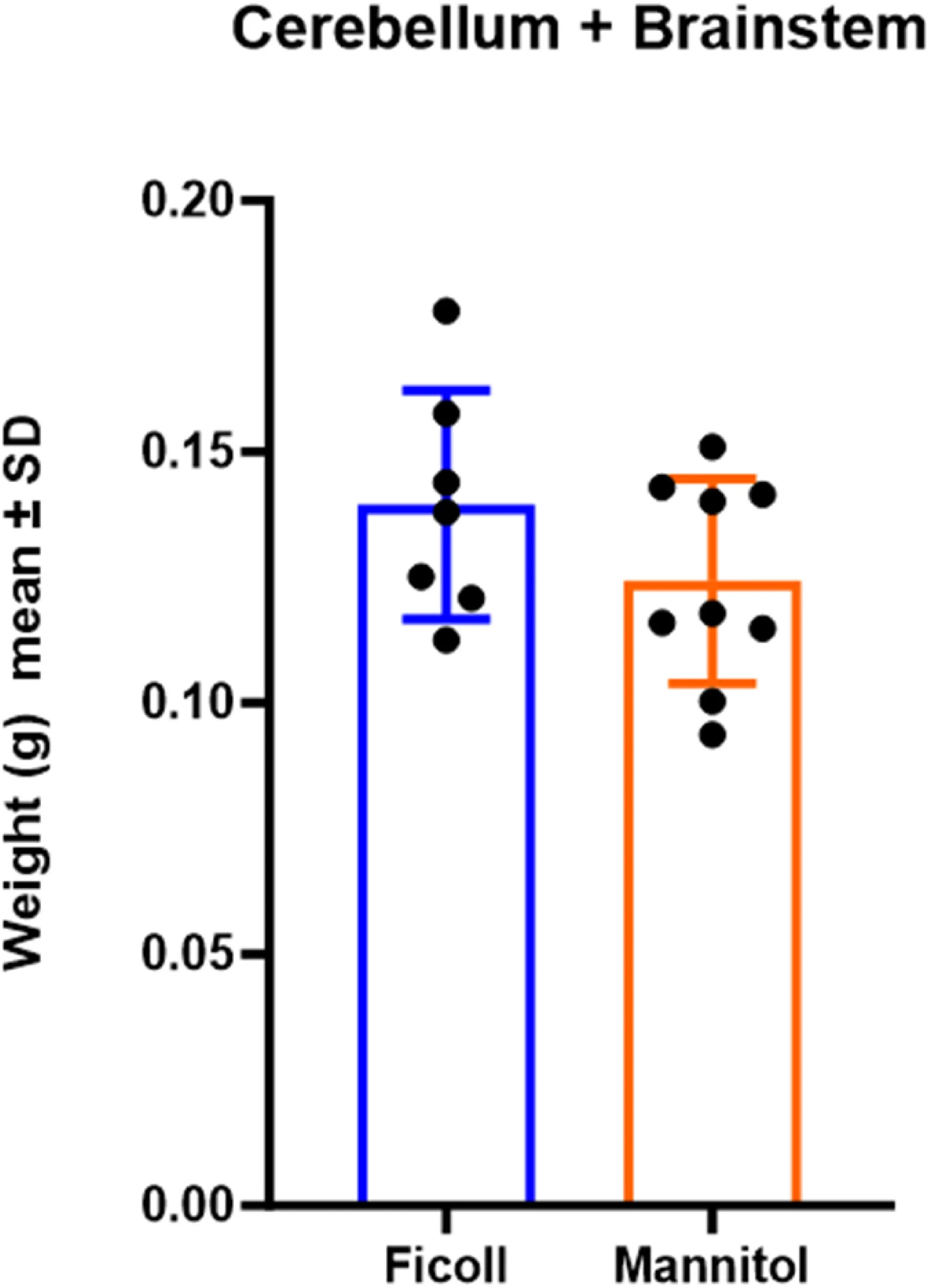
**Comparison of the weight of braintissue after 1h perfusion with Ficoll or mannitol.** The brainstem and cerebellum was isotated after the stop of the perfusion.

### 3.5 Single-neuron recordings in the preBötzinger complex

To evaluate whether mannitol perfusion permits stable cellular-level recordings, we performed juxtacellular recordings from preBötzinger complex neurons in mannitol-perfused WHBPs (n = 4). In one preparation we identified a pre-inspiratory neuron whose discharge preceded and overlapped inspiratory phrenic nerve activity (PNA) bursts (Fig. 4). In addition, we recorded three expiratory neurons and three neurons without clear coupling to the respiratory rhythm (data not shown). Successful juxtacellular recordings have be possible for 3 h after start of the prefusion.

**Fig. 4:**
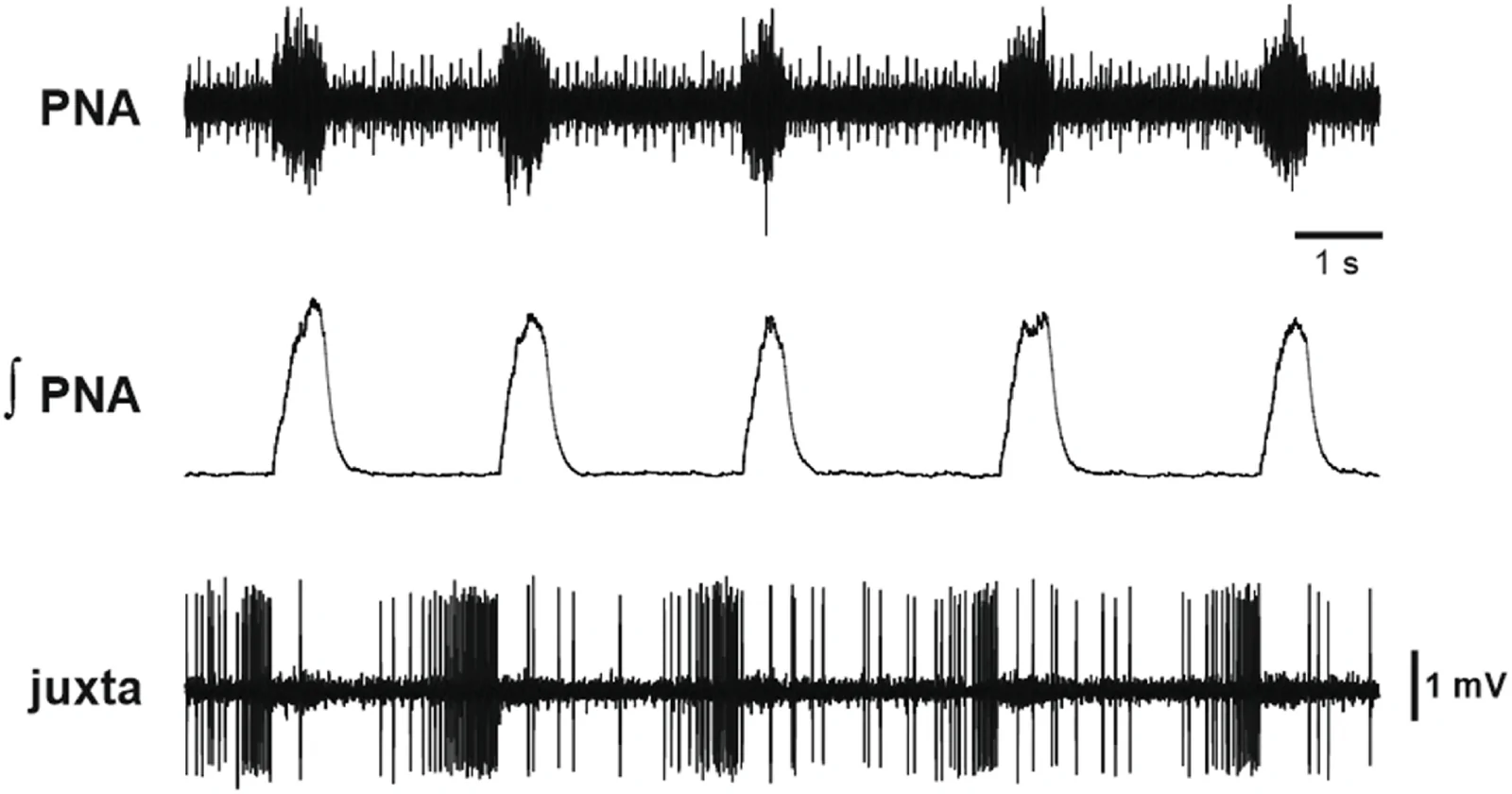
**Successful recording of neuronal action in the WHBP perfussed with 0.85% mannitol.** Representitive juxtacellular recording of a pre-inspiratory neuron from the preBötzinger complex. Upper trace raw: phrenic nerve activity, middle trace:integrated PNA-activity, Lower trace: juxtacellular recording of a pre-inspiratory neuron in the preBötzinger complex.

### 3.6 Post-inspiratory vagus nerve activity

To assess whether post-inspiratory respiratory activity was preserved during mannitol perfusion, we additionally recorded vagus nerve activity in a subset of preparations (n = 5). Clear post-inspiratory vagal discharge was consistently observed under mannitol perfusion (Fig. 5).

**Fig. 5:**
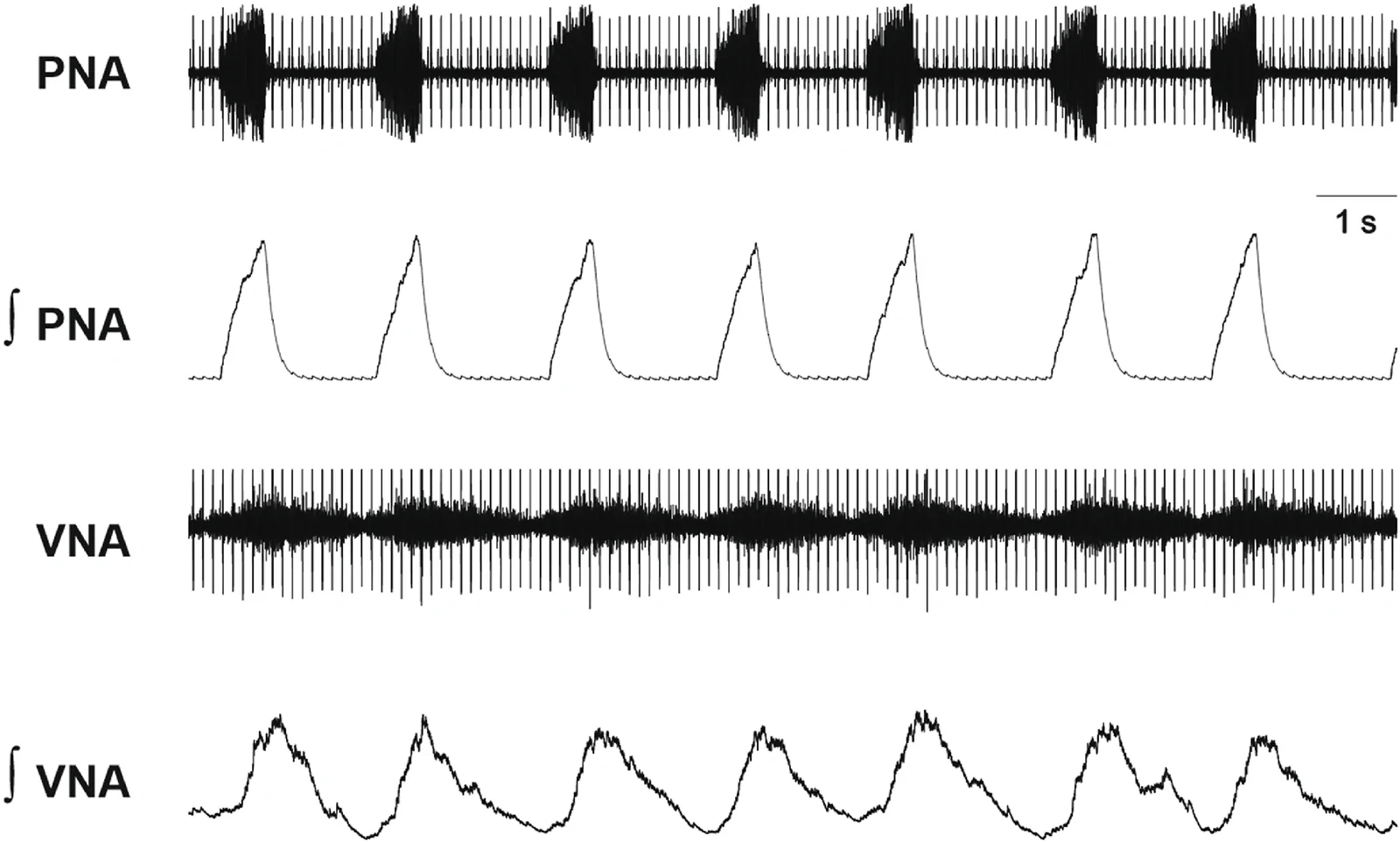
**Post-inspiratory vagus nerve activity in the WHBP perfussed with 0.85% mannitol.** Parallel recordings of vagus nerve activity (VNA) together with the phrenic nerve activity (PNA) with raw and integrated signals (**∫**) in the WHBP during perfusion with mannitol 0.85%.

## 4 Discussion

Replacing Ficoll 70 (1.25%) with mannitol (0.85%) in the perfusate did not compromise viability or core functional output of the mouse WHBP. Both additives supported a stable respiratory motor pattern, with comparable increases in phrenic discharge frequency and preserved amplitude, and neither produced discernible differences in perfusion pressure or brainstem–cerebellum wet weight. Whole-preparation weight increased significantly in both groups, indicating substantial net fluid accumulation, consistent with both additives providing only partial protection against extravasation at the concentrations and perfusion pressures used. These findings indicate that 0.85% mannitol maintains central respiratory network activity and limits gross brain edema to essentially the same degree as 1.25% Ficoll, without either formulation fully compensating for net water efflux.

The central respiratory network behaved similarly under both additives: respiratory frequency rose to a stable eupneic range over the first hour, with closely matched final discharge frequencies, arguing that mannitol does not impair rhythm generation over the time frame studied. Integrated phrenic amplitude declined in both groups as perfusion progressed. Although it remained numerically higher in the mannitol group, the between-group difference was not statistically significant.

Perfusion pressure increased with time in both conditions with no significant between-group difference. Given that 1.25% Ficoll generates only ≈3–4 mmHg oncotic pressure and mannitol's hyperosmolar effect is transient in leaky beds, this shared rise in resistance is physiologically plausible. Overall, functional readouts indicate that mannitol preserves respiratory pattern generation and hemodynamic stability indistinguishably from Ficoll.

Whole-preparation weight gain was greater in the mannitol group than in the Ficoll group. However, the groups had slightly different initial preparation weights, and the larger proportional increase with mannitol should therefore not be interpreted as direct evidence of greater brainstem–cerebellum edema. Consistent with this interpretation, combined brainstem–cerebellum wet weight did not differ significantly between groups after 1 h of perfusion. Thus, the observed difference in whole-preparation weight gain may reflect fluid accumulation in non-neural compartments and/or differences in the distribution of fluid within the preparation; this cannot be resolved from whole-preparation and terminal tissue wet-weight measurements alone.

The critical question for the WHBP is whether the additive protects the brainstem respiratory network, not whether it eliminates extravasation everywhere. The brainstem is supplied via an intact BBB, where permeability to mannitol is very low and most of the transcapillary osmotic gradient is maintained over 1 h (Patlak and Fenstermacher, 1975). Consistent with this, stable respiratory frequency, preserved phrenic pattern, and similar brainstem–cerebellum weights across groups indicate effective brainstem edema control under both conditions.

In contrast, the preparation's extensive non-BBB tissue—vasculature, connective tissue, muscle—equilibrates small solutes rapidly, and Starling forces favour net filtration there. Neither additive is strong enough at these concentrations to abolish extravasation in these leaky beds: Ficoll's oncotic pressure is too small relative to hydrostatic drive, and mannitol's hyperosmolar effect is transient once equilibration occurs, consistent with an overall capillary osmotic reflection coefficient of only ∼0.28 in myocardial tissue and rapid gradient dissipation through 6.9 nm inter-endothelial pores (Kellen and Bassingthwaighte, 2003). This is reflected in the significant time effect on whole-preparation weight regardless of additive. The mechanistic caveat, therefore, is not that mannitol fails to protect the brainstem—as our data demonstrate its efficacy—but rather that global extravasation remains high with both additives. Consequently, tissue weight gain is an inherent characteristic of the preparation itself, rather than a drawback unique to either formulation.

### 4.1 Implications beyond the working heart–brainstem preparation

Although the present study was conducted in the WHBP, the central finding — that a low-cost crystalloid osmolyte can replace a high-cost macromolecular colloid as the primary anti-edema additive in a cell-free arterially perfused preparation — has direct implications for a broad range of isolated and in situ organ perfusion models used across cardiovascular, renal, and neuropharmacological research. In any preparation perfused with a cell-free crystalloid medium, the absence of plasma proteins eliminates the oncotic counterforce that normally opposes capillary filtration, and some form of osmotic or oncotic supplementation is required to maintain tissue water balance. Our data demonstrate that this supplementation does not need to be macromolecular: a crystalloid osmolyte with sufficient osmotic activity and adequate capillary impermeability can provide equivalent functional stability over the timescale of a typical acute experiment. For researchers using isolated perfused heart preparations, where Langendorff protocols commonly employ no oncotic agent at all for short-duration experiments (Bell et al., 2011; Skrzypiec-Spring et al., 2007), mannitol supplementation may offer a straightforward, inexpensive improvement that reduces myocardial edema without altering the ionic composition of the perfusate. For isolated perfused kidney preparations, where albumin is the standard oncotic agent at concentrations of 4–7.5% (Maack, 1980; Schurek and Alt, 1981; Swanson et al., 1981) mannitol is unlikely to be a complete substitute given the much higher perfusion pressures and the specific dependence of glomerular filtration on peritubular oncotic pressure; however, it may serve as a useful supplement in cost-sensitive or pharmacokinetic applications where protein binding of test compounds is a confound. More broadly, the WHBP itself is increasingly relevant to pharmacologists and neuroscientists outside the respiratory physiology community: its combination of intact brainstem circuitry, autonomic efferent outflow, and a beating heart provides a unique platform for testing cardiorespiratory and autonomic drug effects under conditions of controlled perfusate composition, gas tension, and temperature that are unachievable in vivo (Paton et al., 2022). Moreover, the WHBP aligns with the 3Rs framework for the replacement and reduction of animal use in integrative physiological and pharmacological research, making the optimization of its perfusate composition, including the shift to a low-cost, endotoxin-free, batch-stable additive such as mannitol, of practical importance to a growing user community.

## 5 Conclusion

In mannitol-perfused working heart–brainstem preparations, respiratory frequency and phrenic motor output were preserved, with no detectable differences from Ficoll-perfused preparations. Although whole-preparation weight gain was greater with mannitol, combined brainstem–cerebellum wet weight did not differ detectably between groups. Together with its lower cost, defined composition, and lack of batch-to-batch variability, mannitol represents a practical alternative to Ficoll for mouse working heart–brainstem preparation experiments.

## Ethics declaration

This study was conducted in accordance with the ARRIVE (Animal Research: Reporting of In Vivo Experiments) guidelines as well as the following guidelines for animal welfare and/or reporting: German Animal Welfare Act (TierSchG). Ethics committee approval was not required. Mice were killed solely for the purpose of tissue collection in accordance with § 4(3) of the German Animal Welfare Act (TierSchG), which governs killing of animals exclusively for scientific tissue/organ removal and does not constitute an animal experiment under § 7(2) TierSchG. This procedure was registered with the Animal Welfare Officer of the University Medical Center Göttingen (UMG) under file number T22.33.

## Funding

The 10.13039/501100001659German Research Foundation (10.13039/501100001659Deutsche Forschungsgemeinschaft, 10.13039/501100001659DFG) funded this study (Hu797/16-1).

## Declaration of competing interest

The authors declare that they have no known competing financial interests or personal relationships that could have appeared to influence the work reported in this paper.

## Data Availability

Data will be made available on request.

## References

[bib1] Bell R.M., Mocanu M.M., Yellon D.M. (2011). Retrograde heart perfusion: the langendorff technique of isolated heart perfusion. J. Mol. Cell. Cardiol..

[bib2] Cook A.M., Morgan Jones G., Hawryluk G.W.J., Mailloux P., McLaughlin D., Papangelou A., Samuel S., Tokumaru S., Venkatasubramanian C., Zacko C., Zimmermann L.L., Hirsch K., Shutter L. (2020). Guidelines for the acute treatment of cerebral edema in neurocritical care patients. Neurocritical Care.

[bib3] Gan Y., Xu Y., Yu T., Li Y., Jiang X., Jin H., Li H., Zhang Y., Yin P., Kim J.S. (2026). Synchronized imaging of hydrogen peroxide and hydroxyl radical in pyroptosis and epilepsy. Adv. Sci. (Weinh.).

[bib4] Kellen M.R., Bassingthwaighte J.B. (2003). An integrative model of coupled water and solute exchange in the heart. Am. J. Physiol. Heart Circ. Physiol..

[bib5] Levick J.R., Michel C.C. (2010). Microvascular fluid exchange and the revised starling principle. Cardiovasc. Res..

[bib6] Maack T. (1980). Physiological evaluation of the isolated perfused rat kidney. Am. J. Physiol..

[bib7] Magovern G., Bolling S., Casale A., Bulkley B., Gardner T. (1984). The mechanism of mannitol in reducing ischemic injury: hyperosmolarity or hydroxyl scavenger?. Circulation.

[bib8] Patlak C.S., Fenstermacher J.D. (1975). Measurements of dog blood-brain transfer constants by ventriculocisternal perfusion. Am. J. Physiol..

[bib9] Paton J.F. (1996). The ventral medullary respiratory network of the mature mouse studied in a working heart-brainstem preparation. J. Physiol..

[bib10] Paton J.F. (1996). A working heart-brainstem preparation of the mouse. J. Neurosci. Methods.

[bib11] Paton J.F.R., Machado B.H., Moraes D.J.A., Zoccal D.B., Abdala A.P., Smith J.C., Antunes V.R., Murphy D., Dutschmann M., Dhingra R.R., McAllen R., Pickering A.E., Wilson R.J.A., Day T.A., Barioni N.O., Allen A.M., Menuet C., Donnelly J., Felippe I., St-John W.M. (2022). Advancing respiratory-cardiovascular physiology with the working heart-brainstem preparation over 25 years. J. Physiol..

[bib12] Schurek H.J., Alt J.M. (1981). Effect of albumin on the function of perfused rat kidney. Am. J. Physiol..

[bib13] Skrzypiec-Spring M., Grotthus B., Szelag A., Schulz R. (2007). Isolated heart perfusion according to Langendorff---still viable in the new millennium. J. Pharmacol. Toxicol. Methods.

[bib14] Swanson J.W., Besarab A., Pomerantz P.P., DeGuzman A. (1981). Effect of erythrocytes and globulin on renal functions of the isolated rat kidney. Am. J. Physiol..

[bib15] Tacke C., Bischoff A.M., Harb A., Vafadari B., Hulsmann S. (2023). Angiotensin II increases respiratory rhythmic activity in the preBotzinger complex without inducing astroglial calcium signaling. Front. Cell. Neurosci..

